# Factors associated with malaria vaccine uptake in Sunyani Municipality, Ghana

**DOI:** 10.1186/s12936-021-03857-1

**Published:** 2021-07-27

**Authors:** Dennis Tabiri, Jean Claude Romaric Pingdwindé Ouédraogo, Priscilla Awo Nortey

**Affiliations:** Department of Epidemiology and Disease Control, School of Public Health, University of Ghana, Accra, Ghana

**Keywords:** Malaria, Vaccine, Uptake

## Abstract

**Background:**

Malaria continues to be a major disease of public health concern affecting several million people worldwide. The World Health Organization (WHO) started a pilot study on a malaria vaccine (RTS,S) in Ghana and two other countries in 2019. This study aimed at assessing the factors associated with uptake of the vaccine in the Sunyani Municipality of Ghana.

**Methods:**

The study was a cross-sectional study employing a quantitative approach. Stratified sampling technique was used to select respondents. A structured questionnaire was administered to parents/caregivers with children eligible to have taken the first three doses of the malaria vaccine by December 2019. The Child Welfare Clinic (CWC) cards of the eligible children were also inspected. Ordinal logistic regression analysis was done to determine the association between the independent variables and full vaccine uptake.

**Results:**

Uptake of RTS,S 1 was 94.1%. However, this figure reduced to 90.6% for RTS,S 2, and 78.1% for RTS,S 3. Children with a parent who had been educated up to the tertiary level had 4.72 (AOR: 4.72, 95% CI 1.27–17.55) increased odds of full uptake as compared to those who completed secondary education. Parents whose children had experienced fever as an adverse reaction were more likely to send their children for the malaria vaccine as compared to those whose children had ever suffered abscess as an adverse reaction (AOR: 2.27, 95% CI 1.13–5.10). Children with parents who thought vaccines were becoming too many for children had 71% (AOR: 0.29, 95% CI 0.14–0.61) reduced odds of full uptake as compared to those who thought otherwise.

**Conclusion:**

Uptake of RTS,S 1 and RTS,S 2 in Sunyani Municipality meets the WHO’s target coverage for vaccines, however, RTS,S 3 uptake does not. Furthermore, there is a growing perception amongst parents/caregivers that vaccines are becoming too many for children which negatively affects uptake.

## Background

Malaria continues to be a major disease of public health concern affecting several million people worldwide. According to the 2018 world malaria report, about 219 million malaria cases occurred worldwide in 2017. The report further indicates that sub-Saharan Africa represents the region with the highest burden. Ghana, together with nine other African countries and India contributes about 70% of the world’s total malaria burden. Children under 5 years of age are the worst affected. In 2017, 49% and 61% of malaria cases and deaths respectively occurred in this age group according to the same report [1].

In Ghana, 11 million suspected cases of malaria and 5.5 million cases of confirmed malaria were estimated to have occurred in 2018. Approximately 30% of these cases occurred in children under 5 [2]. Malaria is an entirely preventable disease [3]. Several interventions have been introduced over the years in a bid to control malaria. Despite the proven effectiveness of these malaria control interventions, uptake remains low in some parts of the world [1]. The latest intervention to be introduced is the malaria vaccine.

The malaria vaccine known as Mosquirix, RTS,S/AS01, or simply RTS,S is the first vaccine proven to offer partial protection against malaria [3]. The World Health Organization (WHO), however, recommends the use of the vaccine on a pilot basis to inform its broader use. Subsequently, the world body approved a phased introduction of the malaria vaccine in 2019. Ghana, Kenya, and Malawi are the countries involved in this pilot study, in a program called Malaria Vaccine Implementation Programme (MVIP). Selected areas within these countries have been enrolled unto the programme [4].

In Ghana, between 120,000 and 150,000 children in 33 selected districts/Municipalities are targeted to receive the vaccine each year. The vaccine is to be given in four doses at 6, 7, 9 and 24 months of age through the routine Expanded Programme on Immunization (EPI) system [5].

Administration of the first doses of the malaria vaccine in Ghana begun in May 2019. However, news of the introduction of the vaccine was met with viral videos and messages on social media of some persons calling on the public to reject the vaccine. There were claims that the vaccine was unsafe and that Ghanaians were deceptively being used for vaccine trials [6].

The malaria vaccine is envisaged to prevent four out of ten malaria cases and three out of ten malaria deaths [3]. Nonetheless, globally, an estimated 1.5 million children die as a result of missing out on life-saving vaccines yearly [7]. In 2014, 31% out of the 216 districts in Ghana did not achieve the target coverage of 80% for the proxy vaccine (Penta 3), indicating that some children continue to miss out on life-saving vaccines in Ghana [8].

The expected impact of the malaria vaccine is not likely to be seen in Ghana if uptake of the vaccine is not optimum. This study, therefore, sought to assess the factors associated with malaria vaccine uptake in the Sunyani Municipality of Ghana so that uptake can be maximized.

## Methods

### Study area

Sunyani Municipality is one of the 27 administrative districts in the Bono region of Ghana. Its estimated population for 2019 was 151,378. Sunyani is the Municipality’s capital. It lies between Latitudes 70º20′N and 70º05′N, and Longitudes 20º30′W and 20º10′W. It shares boundaries with Sunyani West district to the north, Asutifi district to the south, Tano North district to the east, and Dormaa East district to the west. The Municipality has a total land area of 829.3 square kilometres. One-third of the total land area is not inhabited or cultivated which provides arable land for development [9].

The Municipal Health Directorate (MHD) is in charge of health administration in the district. There are 33 health facilities that provide care to inhabitants of the Municipality. The Municipality is zoned into 34 functional Community-based Health Planning and Service (CHPS) zones under six sub-Municipalities. The sub-Municipalities are Abesim, Antwikrom, Newtown/Baakoniaba, New Dormaa, Sunyani central, and Penkwase. The CHPS zones are demarcated areas for the delivery of health services [9].

Childhood vaccines are delivered at health facilities and outreach points within the Municipality. The outreach points serve to deliver vaccines at the doorsteps of beneficiaries so that physical access does not hinder uptake. The outreach points are organized under the various CHPS zones in the 6 sub-Municipalities. The Municipality is noted for being one of the best in terms of vaccine coverage in the country. It is one of the reasons why it was selected as one of the implementing districts under the MVIP [9].

### Study design and sampling

The study was an analytical cross-sectional study employing a quantitative approach.

Structured questionnaire and an observation checklist were used to collect data on malaria vaccine uptake and related factors. The assessment was done at a point in time giving a snapshot of the situation. Data was collected from parents/caregivers in Sunyani Municipality about themselves and their children on factors related to malaria vaccine uptake.

Cochrane’s formula was used in to calculate the sample size; ${\text{n}} = \frac{{Z^{2} Pq}}{{d^{2} }}$ where n  =  sample size; Z  =  standard normal variate for margin of error; p  =  proportion of children who take RTS,S vaccine; q  =  1-p; d  =  margin of error.

Since this study was on uptake of a new vaccine, an assumed proportion of uptake of 50% was employed; using a margin of error of 5% and a 10% adjustment for non-response, the calculated sample size was 424.

Stratified sampling technique proportionate to size was used in selecting participants from the six sub-Municipalities which served as strata. There are differences in the characteristics of the sub-Municipalities. Employing stratified sampling technique ensured that all sub-Municipalities were represented in the sample. It also allowed comparison to be made across sub-Municipalities.

In determining the proportionate sample size for each sub-Municipality, the formula below was used:

$${\text{A}} = \frac{y}{z},$$

where A is the sampling fraction, ‘y’ is the sample size  =  424, and ‘z’ is the 2019 monthly target coverage for vaccines in Sunyani Municipality  =  505.

The sampling fraction was applied to the monthly target of each sub-Municipality to determine the sample size for each sub-Municipality as shown in Table 1.

**Table 1:** Sample size determination by sub-Municipality (proportionate to size)

Sub-Municipality	Monthly target (x)	Sample fraction × x	Sample size
Abesim	80	$\frac{424}{{505}}$ × 80	67
Antwikrom	53	$\frac{424}{{505}}$ × 53	45
New Dormaa	122	$\frac{424}{{505}}$ × 122	102
Newtown/Baakoniaba	93	$\frac{424}{{505}}$ × 93	78
Penkwase	78	$\frac{424}{{505}}$ × 78	65
Sunyani central	80	$\frac{424}{{505}}$ × 80	66
Total	505		424

Systematic sampling was then used to select respondents from each stratum. A sampling frame was constructed using the Child Welfare Clinic (CWC) registers at the various CHPS zones. The CWC registers contain the official records of each vaccinated child in a particular CHPS zone. The frame contained the names of children who were eligible to have taken the first 3 doses of the malaria vaccine by December 2019, for each sub-Municipality. A sampling interval was determined for each sampling frame using the formula:

$${\text{K}} = \frac{N}{n},$$

where K  =  sampling interval, N  =  the number of children in the sampling frame, and n  =  sample size for the sub-Municipality. Simple random sampling was used to select the first sample by writing the names of the children from one to the sampling interval, folded and mixed up in a bowl. One piece of paper was selected and the name on the paper represented the first sample. Subsequent samples were drawn by adding the sampling interval to the number of the first drawn sample until all samples required for the sub-Municipality were drawn.

The parents/caregivers of the selected children were contacted and those who agreed to be part of the study were interviewed.

### Data collection

Data was collected through the administration of questionnaires to respondents and observation of CWC cards. Parents/caregivers were contacted at CWCs or in their houses depending on where they were available to respond to the questionnaire. Questions centred on socio-demographic factors and other independent variables known to affect vaccine uptake. The other independent variables assessed were: knowledge about malaria vaccine, previous experience with vaccines and vaccination, affordability, and accessibility of vaccines in Sunyani Municipality.

The observation checklist centred on the uptake of malaria vaccine. The CWC card of the children provided this information.

Each questionnaire administration and CWC card observation lasted about 20 min.

To ensure voluntary participation in the study, informed consent was obtained from each parent/caregiver before data collection. None of the parents/caregivers contacted refused to participate in the study, indicating a 100% response rate.

### Data analysis

The data was cleaned and entered into Microsoft excel. Entries were double-checked for errors and corrections made. It was then imported to STATA version 15 and analysed. Frequencies and percentages were generated for sociodemographic characteristics such as age, occupation, marital status, religion, and sex. Median and ranges were generated for the continuous variables.

Uptake of the malaria vaccine was measured as levels: no uptake (no dose received), partial uptake (either first or second dose received), and full uptake (all first three doses received). Ordinal logistic regression analysis was done to determine the association between the independent variables and the level of malaria vaccine uptake. The regression was done first at the univariate level. Independent variables with significant p values at the univariate level were used in a multivariate analysis and the model with the best Akaike’s Information Criterion (AIC) and Bayesian’s Information Criterion (BIC) was selected. For all associations, significance level was set at 5%.

## Results

### Socio-demographic characteristics

A total of 424 parents/caregivers and 424 children participated in the study They were drawn from the six sub-Municipalities in the Sunyani Municipality. The study lasted approximately 10 months.

The median age of parents/caregivers was 29 years (27, 32 years). It ranged from 17 to 45 years. Majority of them (99.3%) were parents with almost all being females (99.5%). Most respondents (60.9%) were married with the rest being either single or cohabiting. Up to 43.2% of respondents had up to secondary education, whiles up to 41.5% of their partners, mostly males had up to tertiary education. Most of the respondents were Christians (75%). Up to 55.2% of respondents were self-employed, whiles 20.5% of respondents were unemployed. However, only 2.6% of their partners were unemployed.

Details of the distribution of the socio-demographic characteristics of respondents are shown in the Table 2.

**Table 2:** Distribution of socio-demographic characteristics of study participants, Sunyani Municipal, 2020

Characteristic (n = 424)	Frequency	Percentage (%)
Sub-Municipality		
Abesim	68	16.0
Antwikrom	45	10.6
Newtown/Baakoniaba	78	18.4
New Dormaa	102	24.1
Sunyani central	66	15.6
Penkwase	65	15.3
Age (years)		
15–19	11	2.6
20–24	46	10.9
25–29	171	40.3
30–34	151	35.6
35 and above	45	10.6
Parent or caregiver		
Parent	421	99.3
Caregiver	3	0.7
Sex		
Male	2	0.5
Female	422	99.5
Marital status		
Single	99	23.4
Married	258	60.9
Cohabiting	67	15.8
Number of children alive		
1–3	378	89.2
More than 3	46	10.8
Educational status		
No formal education	28	6.6
Primary education	141	33.3
Secondary education	183	43.2
Tertiary education	72	17.0
Educational status of partner		
No formal education	14	3.3
Primary education	72	17.1
Secondary education	161	38.2
Tertiary education	175	41.5
Religion		
Christianity	318	75.0
Islam	103	24.3
Traditionalist	3	0.7
Occupation		
Unemployed	87	20.5
Self-employed	234	55.2
Farming	32	7.6
Civil servant	71	16.8
Religion of partner (n = 421)		
Christianity	317	75.3
Islam	100	23.8
Traditionalist	4	1
Occupation of partner		
Unemployed	11	2.6
Self-employed	220	52.3
Farming	40	9.5
Civil servant	150	35.6

### Characteristics of children studied, Sunyani Municipality, 2020

Out of the 424 children, 66.3% were aged 15–16 months. Their ages ranged from 13 to 18 months. The median age was 15 months (15, 16 months). Up to 55.4% of them were males. Almost all of them were delivered at a health facility (94.6%).

Details of the characteristics of children studied are shown in Table 3.

**Table 3:** Distribution of characteristics of children studied, Sunyani Municipal, 2020

Characteristic (n = 424)	Frequency	Percentage (%)
Age (months)		
13–14	79	18.6
15–16	281	66.3
17–18	64	15.1
Sex		
Male	235	55.4
Female	189	44.6
Place of delivery		
Home	21	5.0
Health facility	401	94.6
Unknown	2	0.4

### Uptake of RTS,S

While 94.1% (95% CI 91.4–96.0%) of the children had been administered the first dose of the malaria vaccine, 90.6% (95% CI 87.4–93.0%) had been administered both the first and the second dose with a reduced percentage of 78.1 (95% CI 73.9–83.8%) having been administered all the three doses.

The reasons given for receiving some but not all the doses of the vaccine were: “did not know when the next one was due” − 45.6%, “was not around”, − 23.5%, and “not comfortable with issues surrounding vaccine” − 13.2%. For those who had received no dose of the vaccine, 60% of the mothers said it was their partner’s (husband) decision not to allow their children to be administered the vaccine whiles the rest said it was their own decision to refuse the vaccine.

Distribution of uptake of malaria vaccine in Sunyani Municipality is shown in Table 4.

**Table 4:** Distribution of uptake of malaria vaccine in Sunyani Municipal, 2020

Characteristic	Frequency	Percentage (%)
Level of uptake		
No uptake	25	5.9
Partial uptake	68	16.0
Full uptake	331	78.1
RTS,S 1 uptake		
Yes	399	94.1
No	25	5.9
RTS,S 2 uptake		
Yes	384	90.6
No	40	9.4
RTS,S 3 uptake		
Yes	331	78.1
No	93	21.9
Reason for child receiving some but not all doses of RTS,S		
Did not know when next one was due	31	45.6
Was not around	16	23.5
Not comfortable with side effects	8	11.8
Not comfortable with issues surrounding vaccine	9	13.2
Did not take previous one on time	4	5.9
Reason for child receiving none of the doses of RTS,S		
Partner’s (husband) decision to refuse vaccine	15	60.0
Personal decision to refuse vaccine	7	28.0
Did not know child is eligible	3	12

### Trend of uptake of malaria vaccine in Sunyani Municipality

The uptake of malaria vaccine in Sunyani Municipality shows a declining uptake for the subsequent doses of the vaccine. Whiles uptake for the first dose was 94.1%, it reduced to 90.6% for the second dose and to 78.1% for the third dose. RTS,S 1 and RTS,S 2 uptake met the WHO target of 90% but uptake of RTS,S 3 did not.

**Fig. 1:**
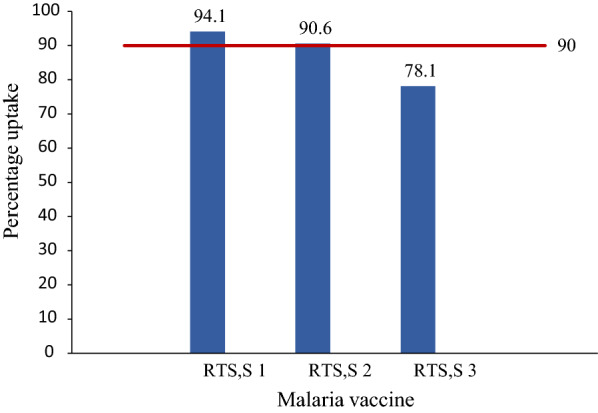
Uptake of malaria vaccine in Sunyani Municipality. Blue bars: uptake (%). Red bars: target

A chart of the uptake of the first three doses of malaria vaccine in Sunyani Municipality is shown in Fig. 1.

### Association between Independent variables and level of malaria vaccine uptake

There was a significant association between the sub-Municipality where one resided and level of uptake. Having a parent who had up to tertiary level education was associated with significantly increased odds of uptake. Details of association between independent variables and level of uptake is shown in Table 5.

**Table 5:** Association between independent variables and level of uptake

Characteristic	Odds ratio	95% confidence interval	P value
Sub-Municipal	0.79	0.69–0.92	0.002
Penkwase (base)	1		
Abesim	2.91	1.29–6.57	0.010
Antwikrom	5.36	1.71–16.79	0.004
Newtown/Baakoniaba	2.28	1.08–4.83	0.031
New Dormaa	1.41	0.73–2.73	0.303
Sunyani central	2.28	1.03–5.08	0.043
Age of parent (years)	1.06	0.82–1.37	0.616
20–24 (base)	1		
15–19	3.41	0.39–29.59	0.266
25–29	1.03	0.48–2.20	0.945
30–34	1.34	0.61–2.94	0.463
35 and above	1.38	0.51–3.73	0.522
Education level of parent/caregiver	1.29	0.97–1.71	0.075
Primary education (base)	1	1	
No formal education	1.52	0.54–4.30	0.432
Secondary education	1.36	0.82–2.26	0.234
Tertiary education	2.37	1.11–5.08	0.026
Education level of partner	1.02	0.78–1.35	0.866
Secondary education (base)	1	1	
No formal education	2.56	0.55–11.89	0.230
Primary education	2.46	1.19–5.07	0.015
Tertiary education	2.08	1.25–3.45	0.005
Number of children alive	0.97	0.46–2.04	0.940
1–3 (base)	1	1	
More than 3	0.97	0.46–2.04	0.940
Marital status	0.80	0.55–1.17	0.254
Cohabiting (base)	1	1	
Single	1.60	0.78–3.27	0.199
Married	1.68	0.91–3.08	0.097
Religion of parent/caregiver	0.73	0.44–1.18	0.205
Traditionalist (base)	1	1	
Christian	2.89	0.23–35.94	0.409
Islam	2.17	0.17–27.63	0.551
Religion of partner	0.73	0.45–1.19	0.204
Traditionalist (base)	1	1	
Christian	1.68	0.16–17.29	0.664
Islam	1.21	0.12–12.85	0.872
Occupation of parent/caregiver	1.34	1.03–1.74	0.027
Unemployed (base)	1	1	
Self employed	1.33	0.76–2.32	0.319
Farmer	2.66	0.85–8.39	0.094
Civil servant	2.21	1.00–4.90	0.049
Occupation of partner	1.13	0.89–1.43	0.305
Self-employed (base)	1	1	
Unemployed	3.24	0.40–26.06	0.268
Farmer	3.07	1.05–9.01	0.041
Civil servant	1.365	0.84–2.23	0.214
Known vaccine preventable diseases	1.01	0.81–1.27	0.899
Up to 3 vpds mentioned (base)	1	1	
No correct vpd mentioned	3.27	1.12–9.56	0.031
4–8 correct vpds mentioned	1.06	0.51–2.23	0.870
Any correct disease but including malaria	1.47	0.86–2.51	0.164
Heard about malaria vaccine	0.64	0.30–1.36	0.249
No (base)	1	1	
Yes	0.64	0.30–1.36	0.249
Where first heard about malaria vaccine	0.86	0.55–1.35	0.510
Friends/relatives (base)	1	1	
CWC	20.22	1.61–253.71	0.020
Health facility announcement	16.78	1.18–239.11	0.037
Radio	39.68	2.14–737.14	0.014
Number of times a child is expected to take the malaria vaccine	1.26	0.67–2.34	0.472
Incorrect number (base)	1	1	
Correct number	1.26	0.67–2.34	0.472
Schedule of malaria vaccine	1.26	0.55–3.04	0.560
Incorrect order (base)	1	1	
Correct order	1.29	0.55–3.04	0.560
Heard about any negative report or issue concerning the malaria vaccine	1.72	1.03–2.88	0.040
Yes (base)	1	1	
No	1.72	1.03–2.88	0.040
Where issue or report was heard	1.38	0.81–2.34	0.232
Radio (base)	1	1	
Friends/relatives	2.75	1.37–5.51	0.004
Health worker	2.28	0.55–9.44	0.255
Other	1.08	0.85–13.60	0.954
Did issue/report prevent or delay vaccine acceptance	8.74	4.32–17.70	< 0.005
Yes (base)	1	1	
No	8.74	4.32–17.70	< 0.005
Given the option of accepting malaria vaccine at CWC	1.15	0.69–1.93	0.591
Yes (base)	1	1	
No	1.15	0.69–1.93	0.591
Are vaccines becoming many for children with the introduction of the malaria vaccine	0.60	0.38–0.97	0.038
No (base)	1	1	
Yes	0.60	0.38–0.97	0.038
Will you recommend malaria vaccine to others			
No (base)	1	1	
Yes	12.61	7.00–22.72	< 0.005
Child ever suffered an adverse reaction following the administration	1.14	0.72–1.80	0.576
No (base)	1	1	
Yes	1.14	0.72–1.80	0.576
Reaction child suffered	0.57	0.41–0.81	0.001
Abscess (base)	1	1	
Fever	3.09	1.56–6.09	0.001
Diarrhoea/vomiting	5.56	0.67–46.00	0.111
Did reaction influence acceptance of other vaccines	1.87	0.56–6.27	0.311
Yes (base)	1	1	
No	1.87	0.56–6.27	0.311
Time taken to reach vaccination centre	1.09	0.69–1.71	0.724
Less than 30 min (base)	1	1	
30–59 min	1.06	0.65–1.74	0.820
1–2 h	1.51	0.18–12.98	0.706
Means of getting to vaccination centre	1.23	0.77–1.99	0.385
Walking (base)	1	1	
Commercial vehicle	1.43	0.85–2.40	0.176
Personal vehicle	0.411	0.07–2.47	0.331
Required to pay any money at vaccination centre	1.24	0.62–2.48	0.544
Yes (base)	1	1	
No	1.24	0.62–2.48	0.544
How to tell when child’s vaccination is due	0.81	0.58–1.12	0.195
Visit clinic monthly (base)	1	1	
Check child’d CWC card	1.57	0.97–2.54	0.064
Told by nurses	2.04	0.80–5.20	0.136
Description of CWC nurses’ attitude	0.95	0.69–1.32	0.779
Excellent (base)	1	1	
Very good	0.50	0.22–1.12	0.092
Good	0.61	0.27–1.40	0.242
Do you think vaccines have long term side effects	1.01	0.33–3.13	0.984
Yes (base)	1	1	
No	1.01	0.33–3.13	0.984

### Multivariate analysis showing association between level of uptake and independent variables

Multiple ordered logistic regression analysis using variables that were significant at 5% in the univariate analysis demonstrated that adjusted odds ratio for uptake per sub-Municipality was not significant.

As compared to secondary education, children with a parent who had been educated up to the tertiary level had an increased odds of 4.72 times of completing uptake. Children with parents/caregivers who thought vaccines were becoming too many for them with the addition of the malaria vaccine had 71% reduced odds of full uptake as compared to those who thought otherwise. This association was significant with a p value of 0.001.

Additionally, children who had suffered fever as an adverse reaction had an increased odds of 2.27 of their children completing uptake as compared to those whose children suffered abscess. Details of the multivariate analysis are depicted in Table 6.

**Table 6:** Multivariate analysis of association between level of uptake and independent variables

Characteristic	Crude Odds ratio	95% Confidence interval	P-value	Adjusted Odds ratio	95% Confidence interval	P-value
Sub-Municipal	0.80	0.69–0.92	0.002	0.82	0.66–1.02	0.076
Penkwase (base)	1	1				
Abesim	2.91	1.29–6.57	0.010	2.21	0.53–9.17	0.276
Antwikrom	5.36	1.71–16.79	0.004	2.01	0.16–26.03	0.593
Newtown/Baakoniaba	2.28	1.08–4.83	0.031	1.22	0.32–4.62	0.770
New Dormaa	1.41	0.73–2.73	0.303	0.36	0.10–1.29	0.117
Sunyani central	2.28	1.03–5.08	0.043	0.98	0.27–3.50	0.971
Education level of partner	1.02	0.78–1.35	0.866	1.02	0.60–1.75	0.936
Secondary education (base)	1	1				
No formal education	2.56	0.55–11.89	0.230	0.93	0.02–31.06	0.970
Primary education	2.46	1.19–5.07	0.015	4.10	1.02–16.47	0.047
Tertiary education	2.08	1.25–3.45	0.005	4.72	1.27–17.55	0.020
Occupation of partner	1.13	0.89–1.44	0.305	1.27	0.84–1.92	0.257
Self-employed (base)	1	1				
Unemployed	3.24	0.40–26.06	0.268	1.74	0.17–17.42	0.637
Farmer	3.07	1.05–9.01	0.041	0.97	0.08–11.06	0.980
Civil servant	1.365	0.84–2.23	0.214	0.61	0.16–2.31	0.464
Vaccines becoming many for children with the introduction of the malaria vaccine	0.60	0.38–0.97	0.038	0.29	0.14–0.61	0.004
No (base)	1	1				
Yes	0.60	0.38–0.97	0.038	0.29	0.14–0.61	0.001
Experience with AEFI	0.57	0.41–0.81	0.001	0.58	0.41–0.83	0.003
Abscess (base)	1	1				
Fever	3.09	1.56–6.09	0.001	2.27	1.13–5.10	0.023
Diarrhoea/vomiting	5.56	0.67–46.00	0.111	6.95	0.69–69.77	0.099

## Discussion

### Uptake of RTS,S

Findings from this study indicated an uptake of 94.1% for RTS,S 1; 90.6% for RTS,S 2; and 78.1% for RTS,S 3. Uptake of RTS,S 1 and RTS,S 2 thus met the target of 90% coverage for vaccines set by WHO [10]. RTS,S 3 coverage, however, did not meet the set target.

There was a reduction in uptake of subsequent doses of the vaccine. This observed reduction is similar to that observed in Senegal, Cameroun, Nigeria, Togo, Congo, and in the Kwabre East district of Ghana [11–16]. The over 90% uptake recorded for the RTS,S 1 and RTS,S 2 indicates that the anti-vaccine campaigns that greeted the introduction of the malaria vaccine did not impact negatively on the uptake of the vaccine in Sunyani Municipality [6]. This may have been so because the messages were largely on social media and did not really seep down to negatively influence parents/caregivers. It may also have been due to effective public education and other community mobilization strategies employed by the Municipality’s health directorate to create awareness about the vaccine when it was introduced.

Out of the 5.9% of children who had not been administered any dose of the vaccine, most (60%) were attributed to a partner’s decision to refuse the vaccine (Table 4). Almost all the respondents were females indicating that it was the fathers who prevented their children from being administered the vaccine. Fathers play a major role in the family and are usually the decision-makers. Those who prevented their children from being given the vaccine may have been influenced by the anti-vaccine campaigns. Fathers are usually not present at CWCs and so are not likely to benefit from education about vaccines which are usually delivered there.

It may also be the case that mothers were unwilling to admit during the interview that they themselves did not want their children to be vaccinated considering the fact that fathers were not around to respond.

The trend of reduced coverages for subsequent doses of the malaria vaccine may be due to poor knowledge of parent/caregivers about the schedule of the vaccines. This could result in parents/caregivers not presenting their children for the subsequent doses on time or not presenting them at all as was the case of 45.6% of respondents (Table 4). Up to 23.5% of children had not been administered all three doses because their parents/caregivers had travelled when they were due. This can be attributed to the fact that not all districts in the country are administering the vaccine (only districts on the MVIP). Therefore, when parents/caregivers travel to these non-implementing districts, their children may not be administered the vaccine at all or on time.

### Factors positively associated with uptake

The findings of higher education and occupation being positive predictors of vaccine uptake are consistent with findings made by Adu, Ofosu, and Mukthar et al*.* [17–19]. Similarly, the findings of Acharya et al*.* [15] of higher education being associated with complete uptake was consistent with findings from this study.

Having a higher educated parent was associated with higher odds of complete uptake both in the univariate analysis and the multivariate analysis (AOR: 4.72, 95% CI 1.27–17.55). This could be because highly educated parents have access to more information about the vaccine and were better placed to understand the implementation programme. Since most parents/caregivers who send their wards for vaccination services are women (99.5%), having a partner who has higher education could mean that as the decision-maker, he is more likely to accept the vaccine. Having a higher education is associated with better occupation, the possible reason why civil servants had higher odds of their children completing uptake when compared.

Additionally, having a parent with primary education was also found to be associated with increased odds of full uptake in the multivariate analysis (AOR: 4.10, 95% CI 1.02–16.47) as compared to having a parent with secondary education. This can be attributed to middle level educated parents being more susceptible to misinformation as compared to lower level educated parents. Whiles lower educated parents may rely on official communication such as health education at child welfare clinics and public announcements, middle level educated parents are more likely to be influenced by the anti-vaccine campaigns which were mainly on social media platforms.

### Factors negatively associated with uptake

The findings of parents/caregivers having the perception that vaccines are becoming too many for their children is unique to this study per available literature reviewed. Those who thought vaccines for children (32.3%) are becoming many had lower odds of completing uptake (AOR: 0.29, 95% CI 0.14–0.61). This could be that parents/caregivers do not see the benefits of the child being vaccinated overriding the potential adverse effect that could occur when the vaccine is given.

Additionally, parents/caregivers who have children who have ever had fever as an adverse reaction (148/223) following immunization had a higher odds of completing uptake as compared to those who had abscess as an adverse reaction (64/223) (AOR: 3.09, 95% CI 1.56–6.09). This could be related to the fact that most parents/caregivers consider fever to be a minor immediate side effect of vaccines as compared to developing an abscess. They were therefore not likely to ‘risk’ going for a new vaccine the safety of which has been questioned.

## Conclusion

Uptake for the first and second doses of the malaria vaccine (RTS,S 1) in the Sunyani Municipality meets WHO’s 90% target. However, uptake of the third dose does not.

Whiles having a higher educated parent is associated with uptake positively, there is a growing perception that vaccines are becoming too many for children and this has a negative impact on uptake.

The Sunyani Municipal Health Directorate and the Ghana Health Service should conduct sustained public education on the malaria vaccine in Sunyani Municipality to further improve upon uptake.

## Data Availability

The datasets used and/or analysed during the current study are available from the corresponding on reasonable request.

## Appendix

See Tables

**Table 7:** Operational definition and scale of measurement for dependent variable

Variable	Operational definition	Scale of measurement	Source of data
Uptake of malaria vaccine	Number of doses of malaria vaccine a child has received	Ordinal	Child’s CWC card
		Full uptake (child has received all 3 doses)	
		Partial uptake (child has received either 1st or 2nd dose)	
		No uptake (child has not received any dose)	

Malaria vaccine is given in 4 doses at 6, 7, 9 and 24 months of age. Only the first 3 doses were considered in this study

7,

**Table 8:** Operational definition and scale of measurement for socio-demographic variables

Variable	Operational definition	Scale of measurement	Source of data
Age	Age in completed years	Ratio	Interview
Sex	Being male or female	Nominal	Observation
Place of residence	Sub-Municipality in Sunyani within which parent/caregiver stays	Nominal	Interview
		Abesim	
		Antwikrom	
		Sunyani central	
		Newtown/Baakoniaba	
		New Dormaa	
		Penkwase	
Marital status	Legal status of relationship with partner	Nominal	Interview
		Married	
		Single	
		Cohabiting	
Religion	Religious denomination	Nominal	Interview
		Christian	
		Muslim	
		Traditionalist	
		Other	
Religion of partner	Religious denomination	Nominal	Interview
		Christian	
		Muslim	
		Traditionalist	
		Other	
Educational level	Highest formal education level attained	Ordinal	Interview
		None	
		Primary	
		Secondary	
		Tertiary	
Educational level of partner	Highest formal education level attained by partner	Ordinal	Interview
		None	
		Primary	
		Secondary	
		Tertiary	
Occupation	What the individual does for a living (brings him/her regular income)	Nominal	Interview
		Unemployed	
		Self-employed	
		Farming	
		Civil servant	
Occupation of partner	What the partner does for a living (brings him/her regular income)	Nominal	Interview
		Unemployed	
		Self-employed	
		Farming	
		Civil servant	
Parity	Number of children alive	Ratio	Interview

8,

**Table 9:** Operational definition and scale of measurement for other independent variables

Variable	Operational definition	Scale of measurement	Source of data
Knowledge about RTS,S	Whether parent/caregiver has ever heard about the malaria vaccine	Binary	Interview
		Yes	
		No	
	Where parent/caregiver first heard about malaria vaccine	Nominal	Interview
		CWC	
		Health facility announcement	
		Radio	
		Friend/relative	
	Knowledge of the number of times a child is supposed to be administered malaria vaccine	Binary	Interview
		Correct number	
		Incorrect number	
	Knowledge of schedule of malaria vaccine administration	Binary	Interview
		Correct order	
		Incorrect order	
Perception of vaccines becoming too many	Parent/caregiver thinks vaccines are becoming too many for children with the introduction of malaria vaccine	Binary	Interview
		Yes	
		No	
Concern about vaccine safety	Heard about any negative issue/report concerning malaria vaccine	Binary	Interview
		Yes	
		No	
Previous experience with vaccines	Whether child has ever suffered an adverse reaction following the administration of a vaccine	Binary	Interview
		Yes	
		No	
Accessibility	Minutes spent in reaching nearest CWC	Ratio	Interview
Affordability	Payment for vaccination services	Ratio	Interview
Perception of quality of vaccination service	Description of CWC nurses’ attitude	Ordinal	Interview
		Excellent	
		Very good	
		Good	
		Bad	
		Very bad	
ANC attendance	Number of times mother attended ANC before delivery of this child	Ratio	Interview
Sex of child	Child being male or female	Nominal	Interview
		Male	
		Female	
Place of delivery	Where child was delivered	Nominal	Interview
		Home delivery	
		Health facility	
Time of uptake	Age (in months) at which child was administered any dose of malaria vaccine	Ratio	Child’s CWC card

9,

**Table 10:** EPI schedule In Ghana (without RTS,S)

Age of administration	Vaccine(s)	Mode of administration
At birth	BCG, OPV 0	Intradermal, oral
6 weeks	Penta 1, PCV 1, OPV 1, Rota 1,	Intramuscular, oral
10 weeks	Penta 2, PCV 2, OPV2, Rota 2	Intramuscular, oral
14 weeks	Penta 3, IPV, PCV 3, OPV 3	Intramuscular, oral
6 months	Vitamin A	Oral
9 months	Measles-rubella (MR) 1, yellow fever	Sub-cutaneous
12 months	Vitamin A	Oral
18 months	MR 2, Meningococcal ‘A’, Vitamin A	Subcutaneous, intramuscular, oral

Source: GHS, 2019

^10^,

**Table 11:** EPI schedule with RTS,S for areas on MVIP in Ghana

Age of administration	Vaccine	Mode of administration
At birth	BCG, OPV 0	Intradermal, oral
6 weeks	Penta 1, PCV 1, OPV 1, Rota 1,	Intramuscular, oral
10 weeks	Penta 2, PCV 2, OPV 2, Rota 2	Intramuscular, oral
14 weeks	Penta 3, IPV, PCV 3, OPV 3	Intramuscular, oral
**6 months**	Vitamin A, **RTS,S 1**	Oral, **intramuscular**
**7 months**	**RTS,S 2**	**Intramuscular**
**9 months**	Measles-rubella (MR) 1, yellow fever, **RTS,S 3**	Subcutaneous, **intramuscular**
12 months	Vitamin A	Oral
18 months	MR 2, Meningococcal ‘A’, Vitamin A	Subcutaneous, intramuscular, Oral
**24 months**	**RTS,S 4**	**Intramuscular**

Source: GHS, 2019

Bolds indicate changes that have occurred in the EPI schedule of MVIP selected areas in Ghana as a result of the introduction of RTS,S

11,

**Table 12:** Frequency distribution of responses

Characteristic	Frequency	Percentage (%)
Sub-Municipality of residence (n = 424)		
Abesim	68	16.0
Antwikrom	45	10.6
Newtown/Baakoniaba	78	18.4
New Dormaa	102	24.1
Sunyani central	66	15.6
Penkwase	65	15.3
Age (years; n = 424)		
15–19	11	2.6
20–24	46	10.9
25–29	171	40.3
30–34	151	35.6
35 and above	45	10.6
Parent or caregiver (n = 424)		
Parent	421	99.3
Caregiver	3	0.7
Sex (n = 424)		
Male	2	0.5
Female	422	99.5
Marital status (n = 424)		
Single	99	23.4
Married	258	60.9
Cohabiting	67	15.8
Number of children alive (n = 424)		
1–3	378	89.2
More than 3	46	10.8
Educational status (n = 424)		
No formal education	28	6.6
Primary education	141	33.3
Secondary education	183	43.2
Tertiary education	72	17.0
Educational status of partner (n = 424)		
No formal education	14	3.3
Primary education	72	17.1
Secondary education	161	38.2
Tertiary education	175	41.5
Religion (n = 424)		
Christianity	318	75.0
Islam	103	24.3
Traditionalist	3	0.7
Occupation (n = 424)		
Unemployed	87	20.5
Self-employed	234	55.2
Farming	32	7.6
Civil servant	71	16.8
Religion of partner (n = 421)		
Christianity	317	75.3
Islam	100	23.8
Traditionalist	4	1
Occupation of partner (n = 421)		
Unemployed	11	2.6
Self-employed	220	52.3
Farming	40	9.5
Civil servant	150	35.6
Age of child in months (n = 424)		
13–14	79	18.6
15–16	281	66.3
17–18	64	15.1
Sex (n = 424)		
Male	235	55.4
Female	189	44.6
Place of delivery (n = 424)		
Home	21	5.0
Health facility	401	94.6
Unknown	2	0.4
Level of uptake of RTS,S (n = 424)		
No uptake	25	5.9
Partial uptake	68	16.0
Full uptake	331	78.1
RTS,S 1 uptake (n = 424)		
Yes	399	94.1
No	25	5.9
RTS,S 2 uptake (n = 424)		
Yes	384	90.6
No	40	9.4
RTS,S 3 uptake (n = 424)		
Yes	331	78.1
No	93	21.9
Reason for child receiving some but not all doses of RTS,S (n = 68)		
Did not know when next one was due	31	45.6
Did not take previous one on time	4	5.9
Not comfortable with side effects	8	11.8
Not comfortable with issues surrounding vaccine	9	13.2
Was not around	16	23.5
Reason for child receiving none of the doses of RTS,S (n = 25)		
Personal decision to refuse vaccine	7	28.0
Partner’s (husband) decision to refuse vaccine	15	60.0
Did not know child is eligible	3	12
Known vaccine-preventable diseases (n = 424)		
No correct disease mentioned	41	9.7
Up to 3 correct diseases mentioned	215	50.7
4–8 correct diseases mentioned	45	10.6
Any correct disease mentioned but including malaria	123	29.0
Heard about malaria vaccine (n = 424)		
Yes	369	87.0
No	55	13.0
Where first heard about malaria vaccine (n = 369)		
CWC	322	87.3
Health facility announcement	28	7.6
Radio	16	4.3
Friend/relative	3	0.8
Number of times a child is supposed to receive the malaria vaccine (n = 369)		
Correct number	73	18.8
Incorrect number	296	80.2
Age order of receiving vaccines (n = 369)		
Correct order	35	9.5
Incorrect order	334	90.5
Heard about any negative report or issue concerning the malaria vaccine (n = 369)		
Yes	225	61.0
No	144	39.0
Where negative issue or report was heard (n = 225)		
Radio	41	18.2
Friends/relatives	168	74.7
Health workers	13	5.8
Other	3	1.3
Negative issue/report heard (n = 225)		
Vaccine is not safe	77	34.2
Children are being used for experiment	140	62.2
Vaccine will affect children’s development	8	3.6
Issue/report prevented or delayed vaccine acceptance (n = 225)		
Yes	46	20.4
No	179	79.6
Given the option of accepting malaria vaccine at CWC (n = 369)		
Yes	247	66.9
No	122	33.1
Vaccines becoming many for children with the introduction of the malaria vaccine (n = 424)		
Yes	137	32.3
No	287	67.7
Recommend malaria vaccine to others		
Yes	357	84.2
No	67	15.8
Reason for recommending vaccine (n = 357)		
It is safe	145	40.6
It protects children against malaria	212	59.4
Reason for not recommending vaccine (n = 67)		
Vaccine does not make any difference	1	1.5
No specific reason	35	52.2
Too many issues surrounding vaccine	2	3.0
Do not have much information on the vaccine	7	10.5
It is not safe	22	32.8
Child ever suffered an adverse reaction following the administration (n = 424)		
Yes	223	52.6
No	201	47.4
Reaction child suffered (n = 223)		
Fever	148	66.4
Diarrhoea/vomiting	11	4.9
Abscess	64	28.7
Did reaction influence acceptance of other vaccines (n = 223)		
Yes	12	94.6
No	211	5.4
Time taken to reach vaccination centre (n = 424)		
Less than 30 min	285	67.2
30–59 min	132	31.1
1–2 h	7	1.7
Means of getting to vaccination centre (n = 424)		
Walking	288	67.9
Commercial vehicle	131	30.9
Personal vehicle	5	1.2
Required to pay any money at vaccination centre (n = 424)		
Yes	50	11.8
No	374	88.2
How to tell when child’s vaccination is due (n = 424)		
Ask friends	12	2.8
Check child’s CWC card	218	51.4
Visit clinic monthly	158	37.3
Told by nurses	36	8.5
Description of CWC nurses’ attitude (n = 424)		
Excellent	54	12.7
Very good	191	45.1
Good	175	41.3
Bad	4	0.9
Vaccines have long term side effects (n = 424)		
Yes	17	4.0
No	407	96.0

^12^

## Abbreviations

AEFI: Adverse Event Following Immunization
ANC: Ante-Natal Care
AOR: Adjusted odds ratio
CWC: Child Welfare Clinic
CHPS: Community-based Health and Planning Service
EPI: Expanded Programme on Immunization
GHS: Ghana Health Service
GVAP: Global Vaccine Action Plan
MHD: Municipal Health Directorate
MVIP: Malaria Vaccine Implementation Program
RTS,S: Malaria vaccine
